# Artificial Intelligence and Robotics in General Surgery: Opportunities and Challenges

**DOI:** 10.7759/cureus.109378

**Published:** 2026-05-21

**Authors:** Sepehr Seifi, Hadi Sahrai, Negin Safari Dehnavi, Fatemeh Amiri, Seyed Mohammad Amin Dashti, Ali Noruzi, Niloofar Taheri, Reza Mosaddeghi-Heris, Ali Seifi

**Affiliations:** 1 Department of Surgery, Arkansas College of Osteopathic Medicine, Fort Smith, USA; 2 Student Research Committee, Tabriz University of Medical Sciences, Tabriz, IRN; 3 Sina Trauma and Surgery Research Center, Tehran University of Medical Sciences, Tehran, IRN; 4 School of Medicine Research Center, Tabriz University of Medical Sciences, Tabriz, IRN; 5 School of Medicine Research Center, Tehran University of Medical Sciences, Tehran, IRN; 6 Department of Psychiatry and Behavioral Sciences, Stanford University, Paolo Alto, USA; 7 Neurology, Tabriz University of Medical Sciences, Tabriz, IRN; 8 Department of Neurosurgery, Division of Neurocritical Care, University of Texas Health Science Center at San Antonio, San Antonio, USA

**Keywords:** artificial intelligence, deep learning, general surgery, intraoperative decision support, machine learning, perioperative care, postoperative complications, preoperative risk prediction, robotic surgery, surgical robotics

## Abstract

Artificial intelligence and robotics are reshaping general surgery across the full perioperative continuum. This narrative review traces the history of surgical robotics from early teleoperated systems developed by NASA and the US Department of Defense to the current da Vinci Xi and SP platforms and examines how AI is now being applied at each phase of surgical care. In the preoperative setting, machine learning models outperform traditional risk scores in predicting postoperative complications, while deep learning applied to computed tomography (CT) and magnetic resonance imaging (MRI) improves tumor detection, lymph node staging, and surgical planning. Intraoperatively, AI-driven phase recognition systems achieve 85-95% accuracy in identifying procedural steps, computer vision tools assess the Critical View of Safety during laparoscopic cholecystectomy, and semi-autonomous robotic systems are beginning to reduce surgeon tremor and automate discrete operative tasks. In the postoperative period, AI-integrated wearable biosensors and electronic health record models enable earlier detection of complications such as sepsis and deep vein thrombosis, while personalized ERAS protocols are refined through continuous data streams. Despite these advances, significant challenges remain, including dataset bias, limited external validation, black-box model opacity, and unresolved questions around data privacy, liability, and regulatory oversight. AI currently functions as a decision-support tool rather than an autonomous actor. Broader clinical adoption will require larger multi-institutional datasets, improved model interpretability, and clear regulatory frameworks.

## Introduction and background

Artificial Intelligence (AI) has found its way into nearly every corner of healthcare, and surgery is no exception. The operating room now generates enormous volumes of structured and unstructured data, from preoperative imaging and laboratory results to intraoperative video recordings and postoperative outcome measures, and AI is increasingly capable of extracting actionable insight from all of it. In some of the most delicate procedures, such as intraocular surgery, AI assistance has already been successfully deployed [1]. Surgery generates enormous datasets ripe for AI analysis. These include preoperative staging data (clinical, laboratory, and imaging); intraoperative data (video recordings and kinematic measurements); and postoperative information (operative times, morbidity and mortality, patient outcomes, and patient-reported outcome measures), which have been used for four decades to capture the patient’s perspective on treatment [2]. Within surgery, AI applications span a broad range: training and simulation, intraoperative decision-making, event and outcome prediction, preoperative planning for complex procedures, postoperative progress monitoring, complication management, and even surgeon credentialing and recertification [3].

The global burden of surgical disease is substantial. Surgical conditions account for 20-35% of the global disease burden and 11% of disability-adjusted life years [4]. Surgical volume, a metric that captures how well surgical needs are being met, highlights the gap: an estimated 160 million additional operations will be needed by 2030 [5]. The Lancet Commission on Global Surgery originally defined surgical volume as procedures per 100,000 population, later refined to “the number of surgical procedures done in an operating theater using any form of anesthesia, per 100,000 population per year” [6,7]. Closing this gap will require tools that extend the reach of existing surgical expertise beyond traditional hospital settings [4,5]. AI and robotic technologies, through telemedicine, remote surgery, and scalable machine learning, offer practical mechanisms for bringing specialist care to underserved populations, positioning them as potential equalizers in global surgical access rather than luxuries of well-resourced systems.

AI has already helped robotic systems improve accuracy, lower risks, shorten recovery times, and speed up a return to work, especially with advances in machine learning and digital imaging [8]. When combined with robotics, AI makes minimally invasive procedures more precise and less prone to complications [9]. Still, challenges around implementation, ethics, and cost-effectiveness remain [10,11]. Deep learning, better access to large datasets, and steady technological improvements have accelerated changes across healthcare. AI-based clinical decision support and robotic surgery are now part of everyday practice in many institutions, representing a major shift in how care is delivered [12]. For any new technology to gain widespread acceptance, it must show clear advantages over existing approaches: better diagnosis or treatment, shorter procedures, lower costs, and improved patient outcomes. It also needs to be scalable, user-friendly, and regulator-approved [8,12].

Robotics and AI are distinct fields, but in surgery their trajectories have converged. Robotic platforms provide the mechanical precision and visualization that modern minimally invasive surgery demands. AI provides the analytical layer, interpreting data, recognizing patterns, predicting risk, and guiding decisions, which makes those platforms smarter. This review treats them together because that integration is now the defining story of surgical innovation. The sections that follow examine this convergence across the perioperative continuum: the historical evolution of surgical robotics, AI applications in preoperative planning and risk stratification, intraoperative decision support and phase recognition, and postoperative monitoring and recovery. We then address the ethical, regulatory, and practical challenges that must be resolved before these technologies can fulfill their clinical promise.

This review examines both the benefits and drawbacks of AI and robotics in the operating room, particularly in general surgery, as well as the challenges ahead and possible paths forward.

## Review

We conducted a narrative review of the development, applications, challenges, and future directions of AI in surgical innovation. The literature search covered PubMed, Scopus, Web of Science, and Google Scholar, targeting peer-reviewed articles, reviews, meta-analyses, and clinical studies published in English through March 2026. The primary search combined terms related to artificial intelligence (including robotic, machine learning, deep learning, neural networks, and computer vision) with surgery, operating room, and perioperative care.

We included articles directly relevant to AI and robotic integration in surgery, historical overviews, clinical applications, ethical considerations, and future directions. Priority went to studies with empirical data, such as accuracy metrics and clinical outcomes, as well as those representing diverse surgical specialties like urology, gynecology, and neurosurgery. We excluded non-English articles, abstracts without full text, non-peer-reviewed sources, duplicate publications, and studies focused on non-surgical AI applications or purely theoretical work without practical implications.

Historical context and evolution of AI in surgery

The idea of using robots for surgery first surfaced in the 1970s, when NASA and the US Department of Defense explored whether teleoperated robots could perform surgery remotely, keeping surgeons away from danger in combat zones or space. The technology at the time wasn't ready, but the concept was laid down [13]. The first robotic platform actually used in human surgery came in 1985, when a Unimation PUMA 200 performed CT-guided neurosurgical biopsies [14]. A team from Imperial College London later modified it for urological and prostate surgeries, creating the four-axis ProBot, the first robotic device designed specifically to remove human tissue [15]. Computer Motion was founded in 1989 and, through a NASA Small Business Innovation Research contract, developed the AESOP arm, an automated endoscope positioner [16]. AESOP received FDA approval for surgical use in 1994. The system evolved into ZEUS, which featured three remotely controlled arms for minimally invasive procedures like endoscopic coronary artery bypass grafting. In 1995, Intuitive Surgical was formed to commercialize robotic surgery technology being developed at SRI International with NIH funding [16,17]. Around that time, a vascular surgeon performed the first telesurgery procedure, an ex vivo intestinal anastomosis, from a control console in a mobile advanced surgical hospital, controlling a surgical unit in a medical forward area surgical team during a combat exercise. The original DoD concept had finally been realized [18]. Several prototypes later, the da Vinci system emerged. Intuitive Surgical began marketing it in Europe in 1999 while awaiting FDA approval in the US. Shortly before going public, the company faced a patent infringement lawsuit from Computer Motion. The issue was finally resolved in 2003 when the two companies merged, phasing out the ZEUS system in favor of da Vinci. While da Vinci went on to dominate the market, the expiration of its patents opened the door to a new wave of competing robotic platforms [16,19]. The da Vinci Xi, launched in 2014, brought better visualization and instrument maneuverability, solidifying its role in complex surgeries. The da Vinci SP system, now authorized in multiple countries, enables single-port procedures that reduce trauma and speed recovery.

In urology, robotic assistance has become especially significant for radical prostatectomy and pyeloplasty. The SP system has shown it can perform complex minimally invasive procedures with skill [20,21]. In gynecology, the technology is used for hysterectomies and myomectomies; studies suggest outcomes comparable to conventional laparoscopy, though operative times vary. The three-dimensional imaging and precise instrument control offer advantages in complex gynecological cases [21]. The da Vinci system has also found a place in cardiac surgery, helping with mitral valve replacement and coronary artery bypass grafting, with minimally invasive approaches that shorten recovery and improve outcomes. In thoracic surgery, it assists with lobectomies and esophagectomies, where better control and visibility mean fewer complications [21,22].

General surgery has adopted da Vinci for cholecystectomy and hernia repairs, where patients benefit from less postoperative pain and better cosmetic results [23]. In orthopedics, robotic-assisted total knee arthroplasty improves implant alignment and positioning. Surgeons can personalize the plan to each patient's anatomy, then use the robot to execute it precisely. The result: better long-term outcomes, less implant wear, and happier patients [24]. Total hip arthroplasty has followed a similar path, with robotics improving preoperative and intraoperative planning, reducing leg length discrepancies, and lowering revision rates [25].

Spine surgery has embraced robotics to improve precision and reduce invasiveness. Surgeons map the spine, then use robotics to place implants and tools with accuracy that means smaller incisions, less tissue damage, and better stability [26]. Pain control is better; recovery is faster. Transoral robotic surgery (TORS) for head and neck cancers allows precise tumor excision while sparing healthy tissue, especially valuable for oropharyngeal malignancies, where preserving speech and swallowing matters [23,27].

The 5G era has pushed telemedicine forward. Remote diagnosis, prompt intervention, and less patient burden, especially for elderly or underserved populations, have become realities. During COVID-19, 5G enabled high-risk patients to receive remote ultrasound and specialist input from hundreds of kilometers away, proving the approach safe and effective [28,29]. In robot-assisted surgery, 5G supports high-speed, low-latency transmission of 4K and 3D imaging, real-time anatomical analysis, and dual-console collaboration for complex or urgent cases, with latency around 271 ms and packet loss under 1% [30]. Telemedicine extends the reach of specialists, while AI-driven deep learning lightens the load: extracting preoperative data, enhancing intraoperative images, recognizing anatomy, and predicting risky events through entropy-based algorithms that flag dangerous tool movements. Postoperatively, AI-driven complication prediction enables personalized recovery pathways, making remote surgery both more efficient and safer [30,31].

AI application in general surgery

General surgery is a highly technical field that depends on manual skill, experience, and careful perioperative integration. Pre-, intra-, and postoperative care all matter to patient outcomes. Over the past few decades, enhanced recovery after surgery (ERAS) pathways have become a focus, aiming to reduce complications and shorten hospital stays [32]. Traditional ERAS protocols apply standardized, population-level pathways to surgical recovery. AI changes this by making ERAS dynamic and patient-specific, using machine learning models and continuous data streams from wearable devices to stratify individual risk, predict recovery trajectories, and adjust perioperative interventions in real time. More broadly, AI has emerged as a promising tool across all three phases of surgical care, preoperative planning and risk stratification, intraoperative decision support and phase recognition, and postoperative monitoring and recovery, working as a decision-support system rather than a standalone diagnostic tool. Advances in robotic surgery, AI-enhanced laparoscopy, and risk assessment all reflect this integration across the full surgical continuum [33].

Preoperative care

Preoperative evaluation matters because surgical procedures carry inherent risks. In complex surgeries like hepatopancreatobiliary (HPB) surgery, extended resections come with high morbidity and mortality. Getting the preoperative assessment right and choosing the right patient for the right procedure is essential for good outcomes. Machine learning (ML) models have shown better predictive accuracy than traditional statistical risk models for postoperative complications. Random forest, gradient boosting, and other algorithms applied to laparoscopic radical gastrectomy, for example, predict morbidity with higher area under the curve values than regression models. These models pull in demographic, laboratory, and comorbidity data to calculate patient risk. But most are built on retrospective data from a limited number of institutions, raising the possibility of bias unless validated on external, multi-institutional data [34,35]. 

Advances made in ML in perioperative care have shown great promise in the realm of risk stratification, prognosis, and decision-making in the context of gastrectomy patients. Preoperatively, using multimodal data comprising clinical factors, laboratory parameters, and imaging characteristics, ML has been able to predict important outcomes in patients undergoing gastrectomy. For example, using ML algorithms to analyze preoperative CT images, quantification of body composition at the L3 level could be performed, with sarcopenic obesity independently predicting reduced survival among gastrectomy patients [36]. Likewise, ML techniques that incorporate 18F-FDG PET/CT metabolic characteristics alongside clinicopathological features have demonstrated excellent capability for predicting the presence of lymph node metastasis, particularly through artificial neural networks and facilitating personalized surgical planning [37]. ML models have been used to predict postoperative outcomes like duodenal stump leakage during surgery, with support vector machine models using clinical data such as tumor features and surgical aspects showing high accuracy (AUC ~0.86) [38]. Moreover, ML-enhanced nomograms for perioperative blood transfusion risk taking into account factors like blood loss, hemoglobin level, nutrition, and metabolism offer practical approaches for better utilization of resources in pre-operative and intra-operative care [39]. These studies underscore the increasingly important need for interpretability and external validation of ML tools in gastrectomies, mirroring similar patterns observed in bariatric surgery, where ML has proven effective in predicting weight trends over long periods, suggesting the utility of data-based precision surgery approaches in gastric surgery [40]. 

Deep learning has also improved preoperative imaging evaluation. Neural networks applied to CT and MRI scans help detect lesions, segment tumors, and localize abnormalities in gastrointestinal and HPB malignancies, rectal adenocarcinomas, lung cancers, and others. These tools assist with surgical planning and margin assessment [41-43]. Perioperative DL-based medical imaging has quickly revolutionized the management of GI cancers because of its ability to provide accurate, evidence-based insights into the characteristics of the tumor itself, the likelihood of metastasis, and outcomes following surgical resection. In colorectal cancer specifically, the use of DL for the detection of lymph node metastasis (LNM) using CT and MR imaging has shown better sensitivity than conventional radiology in several meta-analyses, achieving an AUROC of up to 0.92 [44]. This is complemented by the usage of multimodal DL models, where multiple instance learning algorithms can use histopathology whole slide images along with serum markers of tumors to predict with high accuracy, irrespective of the T stage, while also generating heat maps highlighting high-risk histomorphologic regions [45]. In gastric cancer, convolutional neural networks (CNNs), especially deep residual networks such as ResNet101, have shown superior performance in the detection of early-stage gastric cancer using contrast-enhanced CT images, with an AUC greater than 0.96 and the ability to distinguish between mucosal and submucosal invasion, thus guiding endoscopic versus surgical treatment options [46].

In addition to diagnosing primary tumors, DL-based radiomic workflows have been effectively used to predict complicated perioperative risks such as synchronous peritoneal carcinomatosis through three-dimensional imaging characteristics, resulting in more than 94% accuracy in diagnosis with a superior performance compared to routine interpretations of CT scans [47]. Furthermore, there are multi-task DL models, trained using preoperative imaging data, which predict both peritoneal recurrence and disease-free survival in a consistent manner when validated in both internal and external cohorts and can be utilized clinically to identify patients benefiting from adjuvant chemotherapy [48]. Importantly, interpretability techniques such as Grad-CAM and SHAP have begun to bridge the “black box” gap, highlighting biologically relevant imaging regions and key predictive features, thus facilitating clinician trust and adoption [49]. Taken together, these developments highlight the significant potential of perioperative DL imaging, especially when combined with clinical, molecular, and pathological information, in terms of refining disease staging and developing customized treatment plans in GI oncology [50]. Generative AI (GenAI) is being used to pull together available data from the internet and interact with users. Several GenAI chatbots can already draft surgical manuscripts. They offer an opportunity to improve care, but their output is only as accurate as the data they were trained on, and they can contain errors. That raises ethical questions and underscores the need for human validation [51-53].

Intraoperative care

The main goal of intraoperative AI systems is to improve situational awareness and procedural accuracy. Systems trained to recognize surgical phases help the team stay oriented, potentially reducing cognitive load and improving precision. Early studies show high accuracy in phase detection during laparoscopic surgery, though most rely on small datasets that may limit generalizability [54,55]. Applying AI in surgical analysis comes with technical hurdles. One is data imbalance: surgical videos tend to focus on common procedures, while certain phases are brief and underrepresented, skewing the model. Another challenge is making use of temporal and spatial information, the connections between phases in long videos, or the contact between instruments and tissues in localized images [54]. AI-assisted image analysis has also been used to detect metastatic lesions and pathological abnormalities during minimally invasive surgery. The AiLES laparoscopic exploration system, for instance, uses machine learning to find occult lesions with high sensitivity. But while accuracy looks good, multicenter trials are still needed to confirm its effect on long-term outcomes [56]. AI and ML have had a tremendous impact on intraoperative workflow analysis, especially surgical phase recognition, where there is an effort to recognize phases during surgery for standardization, training, safety, and decision-making purposes. A systematic review revealed that the problem of phase recognition is highly feasible in most models with high levels of accuracy, especially those involving video features and instrument labels; however, most solutions require large amounts of human labeling efforts [57]. In light of this background, it has been shown recently that deep learning algorithms can be used to identify phases in progressively complicated procedures like laparoscopic pancreatoduodenectomy, laparoscopic cholecystectomy, robotic inguinal hernia repair, laparoscopic hemi-hepatectomy, and robotic-assisted esophagectomy, among others, with encouraging results, even in situations with anatomical variations and difficult procedures. Regarding laparoscopic pancreatoduodenectomy, AI succeeded in providing high mean average precision values for both essential and critical steps of the procedure, demonstrating that such technology could be used in highly complex surgeries [58].

Deep convolutional neural networks were used to classify the different stages of laparoscopic cholecystectomy operations with great precision, accuracy, and recall, proving that video-based models are clinically feasible without instrument or time-series data [59]. Edge computing has further improved this technology by using it in real time, which is evident from the case of robotic inguinal hernia repair, where phase prediction was done in real time without excessive delay, indicating that intraoperative AI can move beyond offline analysis toward live workflow monitoring [60]. More recent implementations are in cardiosurgery; however, stress that computer vision systems belong to the relatively advanced AI technologies for use during surgery, yet they remain limited by the data heterogeneity, validation, and integration into routine practice [61]. Similarly, scoping evidence from robotic-assisted esophagectomy shows that intraoperative video analysis and phase recognition are emerging tools for technical assessment, anatomical landmark identification, and intraoperative guidance [62]. Furthermore, the design of a digital assistant system for laparoscopic liver surgeries presents the next stage in this evolution by incorporating phase classification along with organ segmentation and tool detection to achieve real-time safety assessment and quality control with high accuracy, indicating that future surgical AI systems might act as all-encompassing assistants instead of just classifiers [63]. 

AI-based laparoscopic systems have been developed as promising tools that aid in identifying metastases during surgical procedures, specifically in staging laparoscopy and minimally invasive exploration, where surgeons miss metastases due to their elusive nature. The creation of an AI laparoscopic exploration system (AiLES) is a major step forward in this field, exhibiting excellent efficiency in detecting intra-abdominal metastatic foci in late-stage gastric cancer irrespective of lesion size, extent, and location, with a Dice score of 0.76 and the ability to perform in real time and significantly surpassing novice surgeons in the detection of small and subtle lesions, which are critical for staging and treatment planning [56]. Along with such techniques, multimodal ML systems that incorporate DL-based image analysis combined with morphological analysis conducted by the surgeon have proven to yield more accurate differential diagnoses between malignancies and benign tumors in malignant peritoneal metastasis during staging laparoscopy, outperforming the accuracy of other approaches and that of skilled surgeons. Thus, this highlights the value of combining visual and contextual intraoperative data streams [64]. These advancements as a whole illustrate the shift in paradigms towards the use of AI-assisted decision-making during surgery, which may potentially improve cancer staging accuracy through technologies like AiLES and multimodal classification algorithms to aid in lesion detection, decrease diagnostic uncertainty, and avoid unnecessary biopsies. Another important application is in evaluating the critical view of safety (CVS) during laparoscopic cholecystectomy. Deep learning algorithms can assess intraoperative images against CVS criteria, potentially lowering the risk of bile duct injury. Accuracy rates are high, but performance varies with video quality and surgeon experience. In one study of 2,854 images, the mean intersection segmentation was 66.6%, a positive step for surgical safety, but not yet perfect [65,66]. 

Documentation systems using voice recognition and natural language processing, so-called ambient AI, are being developed for operative notes. DAX CoPilot and similar systems aim to cut down on post-surgical documentation time and reduce cognitive burden, letting the team focus on the procedure. But questions remain about data privacy, transcription accuracy, and legal responsibility. A 2022 study found no significant improvement in documentation efficiency or provider productivity with ambient AI scribes in outpatient settings [67]; another study reported a modest reduction in daily documentation time of about 6.89 minutes in ambulatory care [68,69]. The takeaway: Ambient AI may help, but its effectiveness depends on the setting and provider responsibilities. Robotic surgery continues to advance with AI integration. AI can track instruments, filter tremor, and guide imaging. Experimental models have shown some ability to automate aspects of procedures, leading to semi-autonomous systems. Fully autonomous robotic surgery is not yet part of clinical practice, and most surgeons agree that AI should be a tool, not a replacement for their own judgment [11,70,71]. 

Emerging trends in the field of robotic surgery and assistance through AI reveal a trend towards the incorporation of tremor reduction techniques, semi-autonomous tasks, and frameworks for autonomous surgery that will contribute towards precision and reliability. One such challenge in robot-assisted surgery is physiological tremor of the hands, which calls for new solutions and the development of advanced signal-processing and learning-based solutions; notably, a deep learning approach that integrates bidirectional long short-term memory and temporal convolutional networks has exhibited superior performance in predicting tremors by identifying both short- and long-term temporal correlations, resulting in low estimation errors and increased surgical precision within simulated settings [72]. In addition to the application of a data-oriented approach, other types of filter-based methods, including the advanced band-limited multiple Fourier linear combiner (E-BMFLC), have proven to be very effective, resulting in an 8.9% improvement in accuracy in compensating for the tremors and even smaller spatial errors than a millimeter [73]. Apart from motion control, the development of semiautonomous robots, including the smart tissue autonomous robot (STAR), highlights the shift towards task-level autonomy, with vision-based feedback and advanced electrosurgical devices capable of achieving tumor excisions that can be almost on par with an expert surgeon’s work [74]. The emergence of such technology occurs in a larger paradigm of level of autonomy (LoA) in surgical robotics, whereby the technologies in question have transitioned from being completely teleoperated to becoming an integrated system that is able to monitor, plan, select, and execute surgical tasks autonomously [71]. Even though such systems have the potential to minimize variance in surgical results, address staffing issues, and increase worldwide accessibility to quality medical services, there are still some obstacles to overcome, such as a strong perception in a constantly changing operating room setting, tissue discrimination, and thorough validation of the technology in practice. Taken together, all these findings point to an emerging trend of developing smart, semi- to fully autonomous surgical systems where tremor reduction, perception, and decision-making are harmoniously combined [71,72]. 

Postoperative care

ERAS protocols have traditionally applied standardized, population-level pathways to surgical recovery. AI changes this by making ERAS dynamic and patient-specific, using machine learning models and continuous data streams from wearable devices to stratify individual risk, predict recovery trajectories, and adjust perioperative interventions in real time. Postoperative care is becoming more evidence-based, especially with the growth of ERAS protocols. AI algorithms are now being developed to personalize recovery based on predicted risks of complications, prolonged hospital stay, or readmission [75]. Predictive models that combine preoperative and intraoperative data can forecast discharge readiness and identify patients at high risk for early complications. Some models have shown improved prediction of events like sepsis or deep vein thrombosis within the first 48 hours after surgery. Early warning could enable closer monitoring and earlier intervention, potentially reducing morbidity and costs. But many of these models rely on electronic health records, which can be incomplete or inconsistently coded, and practice patterns can influence how well algorithms perform [76,77]. 

Prediction of post-operative complications forms an important part of surgery planning; however, current methods of risk stratification that include the ACS-SR, ASA, and POSSUM scores have linear constraints, limitations of variables considered, and the inability to capture interactions among these, thus leading to poor predictive results [78]. ML, on the other hand, allows for the incorporation of complex, high-dimensional data and non-linear relationships, consistently showing better discrimination than traditional logistic regression techniques in surgical procedures and postoperative complications [78,79]. There is strong evidence from gastrointestinal surgery and general surgery suggesting the high performance of ML algorithms in predicting complications like anastomotic leakage, surgical site infection, pancreatic fistula, and liver failure, with an AUC score more than linear algorithms, for example, 0.90 compared to 0.63 in the case of gastrectomy and 0.87 compared to 0.72 in colorectal surgery [80]. Likewise, applications such as hepatobiliary surgery, cardiothoracic surgery, breast surgery, neurosurgery, orthopedic surgery, and reconstructive surgery have shown how ML algorithms not only enhance the predictive power of risk assessment but also recognize other risk factors associated with each application, which could not be detected through conventional approaches [78]. A meta-analysis confirmed ML’s superiority over logistic regression (mean AUC difference +0.07, p<.001) [79]. Practical application through models like the SMART method for predicting infections after surgery has led to high sensitivity and AUC scores through the use of clinical factors, indicating the translation success of ML (in training validation, 85.3% sensitivity, 74.6% specificity, and AUC=0.89, and during internal validation, with 96.9% sensitivity, 74.1% specificity, and AUC=0.86) [81].

However, even with these benefits, there are obstacles such as the heterogeneity in study design, dependence on data quality, lack of standardization in outcomes, as evidenced in studies involving laparoscopic cholecystectomy, and issues related to interpretability, external validation, and generalizability [82,83]. In essence, machine learning is the paradigm shift from static risk calculators based on population statistics towards dynamic models that use data and continuously learn to predict outcomes for specific patients, which could optimize patient selection and surgery, with perioperative management playing an important role, provided that appropriate validation studies are done [78,80,83]. 

Recent advances extend AI beyond EHR-based models. Wearable biosensors now track heart rate, respiratory rate, oxygen saturation, motion, and other physiological parameters. AI can analyze these data streams to spot potential complications early. These technologies support patient safety through remote surveillance. For now, though, AI functions more as a member of the care team than as an independent decision-maker. Ethical questions about data privacy, transparency, and accountability remain under active discussion [84,85]. In terms of recovery, ERAS has revolutionized post-surgical care through its adoption of evidence-based protocols for surgical care, which have led to decreased complications, faster recovery times, shorter hospitalization periods, and greater patient satisfaction, showing a 50% decrease in postoperative complications with strict compliance with ERAS guidelines.

The development of ERAS has moved towards making the process personalized by linking it with digital health technologies and AI, allowing for individualized recovery processes [86]. AI models may classify patient risks, predict their recovery, and customize perioperative treatments, whereas constant streams of data provided by wearable technologies, electronic medical records, and mobile applications help adjust postoperative care management, including pain relief and monitoring for possible complications [86]. Continuous physiologic monitoring through wearable devices, especially with AI-powered biosensors and smartwatches, is capable of measuring vital signs such as heart rate, oxygen saturation, and physical activity, allowing for timely recognition of complications like hypoxia, arrhythmias, and unstable hemodynamics, thus overcoming the challenges associated with periodic monitoring and possibly minimizing ICU admissions and hospital stay [87]. The combination of wearable devices with electronic patient-reported outcomes (ePROs) in thoracic surgery has demonstrated a strong correlation with clinical measures and improved sensitivity for identifying outlier patients, providing a dependable platform for postoperative monitoring of patients along ERAS protocols [88].

Similarly, wearable-assisted activity monitoring that is consistent with ERAS mobilization strategies has helped provide concrete information about patterns of recovery, such that activity following surgery has been negatively correlated with complications, despite the lack of consistency in outcome improvements. Randomized clinical data from colorectal cancer surgery using the wearable ePM/ep pod show a more refined understanding of the personalization of ERAS protocols, as real-time activity feedback does not appear to have reduced the 30-day comprehensive complication index (CCI) significantly but allows for accurate quantification of activity and showed an independent inverse relationship between increased activity on postoperative day three (215 minutes of activity) and complication severity (β = −0.025) [89]. Thus, while wearable feedback alone may not directly improve outcomes, objective activity tracking can identify recovery thresholds that help individualize ERAS goals.

**Figure 1:**
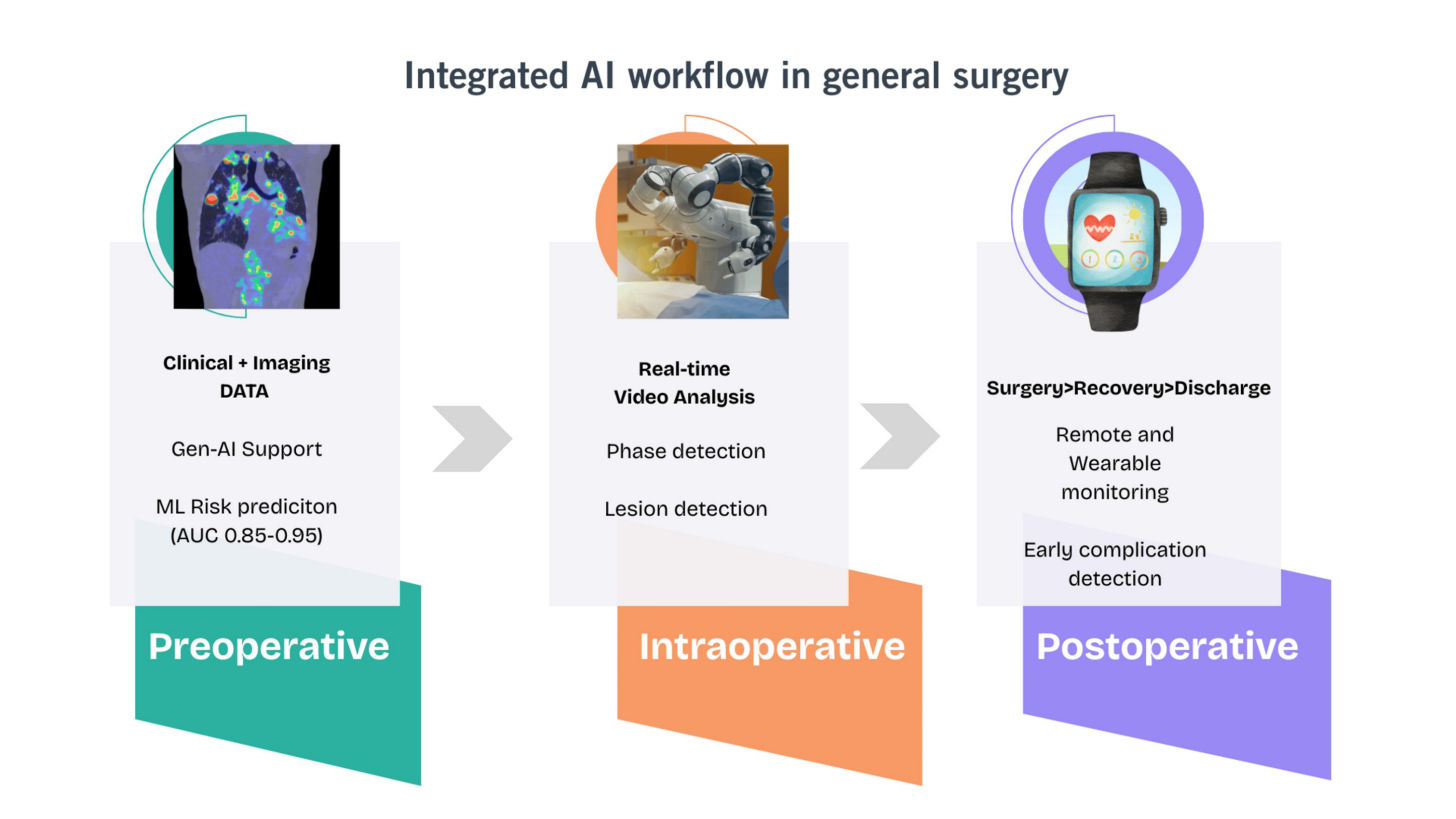
Integrated AI workflow in general surgery illustrating decision-support tools across the perioperative continuum, with key performance metrics and limitations derived from 2023–2026 studies. AI remains assistive, enhancing rather than replacing surgeon. Image is created by author Dr. Seifi via Canva.com

Taken together, AI integration into general surgery is promising and has reached clinical application. But it is not yet a standalone, fully reliable presence in the operating room. Dataset bias, external validity issues, and ethical concerns still matter. AI is unlikely to replace surgeons anytime soon, but it can help them work with greater accuracy, better predictions, and sharper image analysis (Table 1, Figure 1).

**Table 1:** Summary of key AI applications in general surgery across perioperative phases.

Phase	Key AI Tool/Example	Reported Benefit / Metric	Main Limitation / Bias
Preoperative	ML risk models	AUC 0.85–0.95 vs. 0.70–0.80 traditional	Retrospective; single-center bias
Preoperative	DL pelvic segmentation	DSC 0.85–0.95; 30–50% fewer manual edits	Protocol variability; homogeneous data
Intraoperative	Phase recognition	85–95% accuracy; 20–30% cognitive load reduction	Small datasets; 10–15% generalizability drop
Intraoperative	DAX CoPilot	50–70% documentation time savings	Privacy/legal risks; transcription errors
Postoperative	ERAS prediction & wearables	AUC 0.80–0.90; 15–25% morbidity reduction; 2–4h earlier sepsis detection	EHR incompleteness; coding variability

Comparative analysis: bridging surgical domains

Comparing AI use across general surgery fields reveals both synergies and gaps. General surgery benefits from efficiency and scalability, especially in high-volume procedures like hernia repairs [90,91]. The large patient numbers create massive datasets, which are critical for developing effective machine learning algorithms. Robotic systems combined with sophisticated AI models, such as those that improve phase identification during robotic-assisted inguinal hernia repair, function as intraoperative co-pilots. These systems rely on computer vision for real-time anatomical detection, suture analysis, and automation of repetitive tasks. The result is optimized operative duration, fewer intraoperative complications, and lower recurrence rates through standardized mesh positioning and tensioning [92]. A systematic review of 152 studies emphasized AI's crucial role in laparoscopic and robotic general surgery, particularly in detecting the "critical view" during minimally invasive procedures, a form of vigilance that reduces injuries from human cognitive fatigue [93].

Despite differing clinical priorities across specialties, transferable innovations are emerging. Da Vinci continues to rule the market, as newer surgical robots have so far only reached around 80% of its capability. While da Vinci dominates general surgery because of its dexterity in the abdominal space, the technology is now informing next-generation neurosurgical robots. Advanced tremor filtering and micro-scale, precise resections are common themes that are driving platforms that will benefit both specialties [23].

Machine learning models for preoperative risk prediction may be the most transferable assets. These models rely on common clinical variables, patient comorbidities, socioeconomic information, and procedural details to predict complications or mortality with accuracy that consistently beats traditional tools like the ASA physical status classification. Transfer learning plays a key role here, allowing models trained on large general surgery datasets to be adapted for the more specialized neurosurgical environment, improving predictions of 90-day outcomes or postoperative adverse events even when primary neurosurgical data is limited [83,94-96]. Computer vision for anatomical marker identification and augmented reality overlays also provides a technical common language between specialties. These technologies help position a hernia mesh precisely and navigate deep cranial approaches equally well [97]. Still, gaps remain. Direct comparative studies evaluating the same AI tools across different surgical specialties are largely absent, limiting our understanding of generalizability, cost-effectiveness, and the modifications each specialty requires. The potential runs both ways: efficiency-driven innovations from general surgery could improve neurosurgery, while the precision demands of neurosurgery could strengthen AI applications across the entire surgical field.

Challenges and ethical considerations

As AI integrates more deeply into surgery, it brings not only opportunities but also real challenges and ethical dilemmas that demand careful attention. Algorithmic bias stands out as a major concern. AI systems trained on non-diverse datasets risk perpetuating inequities, failing to represent underrepresented groups in risk analysis or imaging, whether for hernia repairs or brain surgery [98]. Beyond bias, the "big data" needs of AI conflict with long-held principles of patient confidentiality. Data privacy is complicated by regulations like HIPAA. Large-scale model training using perioperative data, intraoperative video, and imaging carries a high risk of accidental breaches. Surgical data often contains unique anatomical characteristics that could serve as biometric identifiers, demanding high standards of de-identification and more transparent consent processes. Ensuring that patient information advances medical science without violating privacy remains one of the deepest structural challenges in the digital surgery era.

Transparency gaps in "black-box" models are another barrier to clinical adoption. Many deep learning models reach highly accurate results without providing a clear rationale for their reasoning. That lack of clarity makes it hard for surgeons to trust and understand AI recommendations, and it creates accountability problems. When complications happen, it's unclear whether the algorithm developer or the surgeon who relied on the recommendation bears liability. Informed consent becomes more complicated, and maintaining human agency matters: patients must be told about AI's role in their care, and surgeons must feel empowered to override algorithmic recommendations when clinical judgment dictates [99,100]. Barriers like validation and post-market surveillance delay deployment, though they are necessary for safety. Surgeons generally value AI's ability to reduce errors and support decision-making, but ethicists and patients alike raise concerns about over-reliance and the need for human oversight [101].

In the author's opinion, cost-effectiveness remains an under-addressed dimension of AI and robotic integration in surgery. The high upfront investment required for robotic platforms, ongoing maintenance costs, and the infrastructure needed to support AI systems present significant barriers to adoption, particularly in resource-constrained and low-income settings [10,11]. Comparative health economic analyses are sparse, and most published studies report clinical outcomes without accompanying cost data, making it difficult for health systems to justify adoption on financial grounds alone [23]. Future research should prioritize economic evaluations alongside clinical trials to ensure that the benefits of AI and robotics in surgery are accessible beyond well-resourced academic centers. Mitigation strategies include using ethical checklists in guidelines, curating diverse datasets, developing explainable AI, and building multidisciplinary frameworks that emphasize inclusivity and transparency [102]. These approaches, grounded in empathy for stakeholder concerns, pave the way for responsible AI use and meet the demands of ethical assessment.

Future directions

We are still far from a world where AI-driven decisions are autonomously executed by robotic devices. What contemporary technical progress has genuinely achieved is catalyzing the digital transformation of healthcare. Digitalization moves healthcare processes onto electronic platforms and allows data to be processed and analyzed by algorithms [103]. That shift enables systematic data collection, which in turn generates feedback to improve future outcomes. Looking ahead, integrating AI with virtual and augmented reality, along with next-generation autonomous robotic systems, could significantly change clinical practice. These innovations remain largely experimental, but research is exploring how they might become part of routine care [12]. Combining AI with multi-omics analysis, robotic navigation, super-resolution imaging, nanoparticle technology, and internet-based remote technologies would greatly expand surgical applications. These advances will enhance surgical precision, safety, and efficiency. Through interdisciplinary collaboration and ongoing innovation, AI is poised to play an increasingly central role in global healthcare systems, serving a diverse range of patients [104]. More than 520 AI/ML algorithms have already been authorized, most in radiology and oncology [105], but surgery is next.

Augmented reality is proving useful in spine tumor surgery, extending beyond spondylectomy and osteotomy. During resection of a grade 1 presacral ganglioneuroma, AR was noted to minimize exposure size and improve resection precision [106]. Studies show AR can effectively visualize tumor outlines and adjacent structures with minimal registration error, pointing to future applications in tumor surgery [107,108]. Mixed reality navigation has been investigated for fracture care: in guidewire placement, AR-assisted methods showed better precision, less radiation exposure, and shorter insertion time because line-of-sight obstructions were reduced [109]. In transforaminal lumbar interbody fusion (TLIF), AR has been shown to improve workflow and reduce errors, as demonstrated in a study of ten patients [108,110]. Future developments may include generative AI for personalized extended reality training, adapting scenarios to the surgeon's experience and case complexity. Highly customized surgical blueprints based on patient-specific anatomy are also likely to become standard. AI-driven simulations will allow virtual practice of complex procedures, reduce patient risk, and help address global shortages in surgical expertise [110]. AI is ushering in a transformative era in surgery, marked by greater precision, personalization, and better outcomes. Through advanced analytics and machine learning, AI can process complex medical images, support real-time decision-making, and enable predictive modeling. These capabilities let surgeons perform complex procedures with increased accuracy and confidence. AI serves as a valuable tool in preoperative planning, intraoperative guidance, and postoperative care, elevating surgical standards. Its role in surgical education and training enriches learning experiences and enables objective skills assessment, helping train the next generation of surgeons. As the technology matures, it is positioned to become a cornerstone of surgical innovation [111].

**Figure 2:**
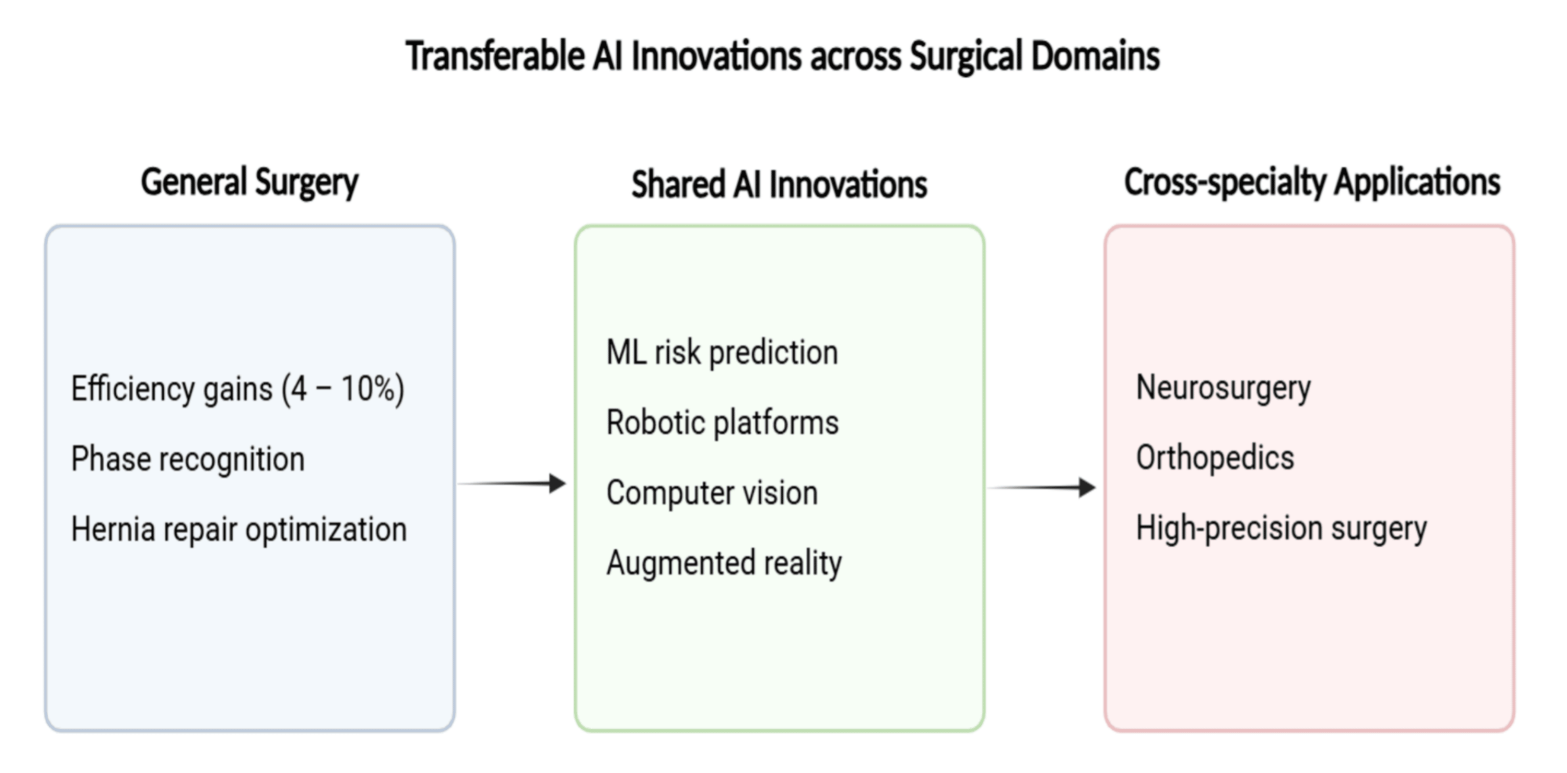
Synergies and transferable AI innovations in general surgery, highlighting shared machine learning models, robotic platforms, and imaging tools with quantifiable benefits and remaining gaps in validation and generalizability. Created in BioRender. Taheri, N. (2026) https://BioRender.com/y3doeq8

In the future, AI systems may offer a superior alternative to human-led interventions, potentially becoming the gold standard, perhaps even eliminating the need for human supervision or control [112]. The next conceptual frontier is artificial general intelligence autonomous systems that match or exceed human intelligence, with consciousness, sentience, and agency. The Turing test, originally designed to determine whether computers could behave indistinguishably from humans, has been adapted into a diagnostic tool (modified Turing test) to provide a quantitative framework for evaluating next-generation AI capabilities. Edge computing and machine learning will enable real-time analysis at the point of care, turning raw data into actionable intelligence without delay. But this raises profound ethical and legal questions: if a robot with agency errs and causes harm, who is accountable? Analysis of legal and ethical frameworks around this scenario sorts responsibility into accountability, liability, and culpability [113,114]. An autonomous robot may bear blame and liability, but it cannot be deemed responsible in a legal sense; it cannot be punished by a court because it lacks civil freedoms. Culpability must fall on those involved in creating or operating the robot. That leads to questions about whether humans should ever be fully excluded from the process. If a surgeon remotely commands a robot and loses signal or experiences a malfunction that injures a patient, liability discussions become essential [114]. Bibliometric analysis from 1992 to 2024 shows robotic surgery has become a rapidly growing field of inquiry. The increase in academic output reflects growing interest and concern within the international scientific community [115] (Figure 2).

Autonomous surgery requires strong mechanism design, path planning, control algorithms, perception, and localization. Current work remains preliminary. Studies are exploring autonomous endoscopic camera navigation using multi-instrument tracking algorithms to achieve ideal visual perspectives [116,117]. Methods are also being investigated for autonomous tissue retraction to improve organ exposure and access [117,118]. Many challenges remain, but progress continues.

Machine learning, a branch of artificial general intelligence, excels at finding patterns in large datasets. This capability opens new avenues for research and innovation in surgical training. Machine learning can deliver instantaneous forecasts of surgical outcomes, improving training and assisting surgeons during procedures [119]. AGI efforts are focusing on autonomous surgical robots that use computer vision and deep learning for object detection and classification, though challenges persist in real-time, large data collection, and sharing [120].

As AI and robotics become more autonomous, ethical questions multiply. Current robotics principles emphasize human-robot interaction and preventing human injury, but frameworks will be needed to regulate interactions among multiple artificial entities. Some have proposed AI legislation that would recognize a sentient robot's inherent right to dignity and equitable treatment while protecting against abuse by other AI technologies [114,121]. The legal use of AI in surgery requires a multidisciplinary approach involving engineers, clinicians, biotechnologists, policymakers, and regulators. Using AI robots in surgery could benefit surgeons, but it must be approached with care.

Robotic certification is increasingly becoming a formal requirement for resident progression in many general surgery training programs [122]. While this reflects the growing clinical relevance of robotic platforms, it also introduces a subtle but important source of institutional bias. Market-leading systems such as da Vinci have intensified educational outreach to residents, with platform-specific online modules, simulation curricula, and credentialing certificates becoming the default pathway in many programs. This early entrenchment in training normalizes a single platform's workflow and limits resident exposure to alternative or emerging robotic technologies. Surgeons trained exclusively on one system may carry those practice patterns and the research questions they generate into their independent careers, compounding the algorithmic bias already noted with non-diverse training datasets. Ensuring that robotic education remains platform-neutral and evidence-driven, rather than industry-shaped, is an ethical responsibility that training programs and professional societies must address proactively.

## Conclusions

Artificial intelligence and robotics are increasingly finding practical applications in general surgery. Machine learning models have shown improved ability to predict postoperative complications compared to traditional risk scores, while deep learning has enhanced tumor detection on cross-sectional imaging. In the intraoperative setting, AI systems can now identify surgical phases and assess key safety steps, such as the Critical View of Safety during cholecystectomy. In addition, wearable devices integrated with AI analytics may allow earlier detection of complications such as sepsis and venous thromboembolism.

Despite these advances, several limitations should be considered. Much of the current evidence is based on retrospective and single-center studies, which may limit generalizability and introduce bias. Multi-center clinical trials will provide stronger support. Many AI models also lack transparency, making their decision-making processes difficult to interpret and potentially limiting clinical adoption. Furthermore, issues related to data privacy, particularly with the use of intraoperative video, remain unresolved. At present, AI is best viewed as a tool to support, rather than replace, the surgeon. Technologies such as augmented reality and semi-autonomous systems are likely to be integrated into practice sooner than fully autonomous surgical platforms. Future progress will depend on the development of larger, multi-institutional datasets, improved model interpretability, and clearer regulatory guidance. Careful, evidence-based implementation will be essential to ensure these technologies are used safely and effectively in clinical practice.
